# Detection and Enhancement of Ketocarotenoid Accumulation in the Newly Isolated Sarcinoid Green Microalga *Chlorosarcinopsis* PY02

**DOI:** 10.3390/biology7010017

**Published:** 2018-02-12

**Authors:** Peelada Cherdchukeattisak, Paul D. Fraser, Saul Purton, Thanyanan Wannathong Brocklehurst

**Affiliations:** 1 Department of Biology, Faculty of Science, Silpakorn University, Nakhonpathom 73000, Thailand; cherdchukeattis_p@silpakorn.edu; 2 School of Biological Sciences, Royal Holloway University of London, Egham TW20 0EX, UK; p.fraser@rhul.ac.uk; 3 Institute of Structural and Molecular Biology, University College London, London WC1E 6BT, UK; s.purton@ucl.ac.uk

**Keywords:** natural colourant, carotenoid, ketocarotenoid, canthaxanthin, nitrogen deprivation, *Chlorosarcinopsis*

## Abstract

The sarcinoid alga PY02 is a newly isolated soil alga native to western Thailand. In this study PY02 is described, the carotenoid profile of the green and red forms of the algal cells are compared, and the effect of nitrogen reduction and media volume on ketocarotenoid production are reported. Partial sequences of the genes from elongation factor Tu (*tuf*A) and 18S rRNA reveal that the alga is from the *Chlorosarcinopsis* genus. Growth studies demonstrated that *Chlorosarcinopsis* PY02 is capable of photoautotrophic, heterotrophic and mixotrophic growth. A gradual change in colony colour from green to red was observed over a period of four weeks under mixotrophic conditions. Pigment analysis of lyophilized red cells using ultrahigh performance liquid chromatography (UPLC) with Photo Diode Array Detection (PDA), showed for the first time that an alga from the genus *Chlorosarcinopsis* is capable of producing ketocarotenoids such as adonixanthin and 3-OH-echinenone, with canthaxanthin as the dominant pigment. Interestingly, a reduction of nitrogen in the medium exerts a positive effect on the rate of colour change from one month to less than seven days. Enhancements of the canthaxanthin content from 520 to 1504 or 1427 µg·gDW^−1^ were detected under 50% and 10% nitrogen content, respectively. An increase of 16% in biomass production of PY02 was unexpectedly detected from a 50% nitrogen reduction under mixotrophic culture. Notably, in liquid mixotrophic media with volumes of 15, 30 and 60 mL, the lowest volume produced a significantly higher biomass and canthaxanthin content.

## 1. Introduction

Ketocarotenoids, such as astaxanthin and canthaxanthin are commercially important carotenoids. These pigments have applications in the nutraceutical, pharmaceutical and cosmetic industries, and are also used in the aquaculture and poultry industries [1,2,3]. Ketocarotenoids not only exhibit powerful antioxidative properties [1], but also help to protect against a wide range of human diseases including UV light-induced tumours [2,3], benign prostatic hyperplasia, prostate and liver tumours [4,5,6], atherosclerosis, and cardiovascular disease [7]. They also strengthen the body’s immune system [8,9,10], and have anti-ageing [11] and anti-inflammatory properties [10,12]. In animal feed, ketocarotenoids (commonly canthaxanthin, phoenicoxanthin and astaxanthin) are added as feed supplements for colouring and health benefits. They enhance the yolk colour in laying hens through their ability to be digested and accumulated in the yolk [13]. Additionally, astaxanthin and canthaxanthin have been shown to improve the growth of farmed fish and shrimp [14,15]. In nature, however, there are only a few organisms that have the ability to synthesize and accumulate ketocarotenoids; principally certain bacteria [16], yeast [17] and algae [18].

Green microalgae are the largest group of organisms that possess the ability to synthesize and accumulate ketocarotenoids. Astaxanthin and canthaxanthin have been found in many species of green microalgae, such as *Haematococcus pluvialis* [19], *Neochloris wimmeri* [20], *Chlorella zofingiensis* [21], *Coelastella triolata* [22], *Scenedesmus* spp. [23] and *Dactyllococcus* sp. [24]. These pigments function as secondary carotenoids in algae and as such, are not involved in photosynthesis. Rather, they localize outside the chloroplast and protect the cells against harsh environments [25]. Algae accumulate high amounts of ketocarotenoids in response to stress conditions such as high light intensity, high salinity and nutrient deprivation [24,26,27,28]. This mechanism also affects their physiology: e.g., some species transform their cells into cysts or a resting stage [29]. Characteristically, the feature of almost all ketocarotenoid-producing algae is that their visual colony colour turns from green into a distinctive orange/red colour when ketocarotenoids accumulate in their cells [22,23,24].

The sarcinoid green microalgae of the genus *Chlorosarcinopsis* are ubiquitous organisms commonly distributed in terrestrial and freshwater environments [30]. The accumulation of secondary carotenoids in this genus, which is noticeable by the change in colony colour from green to a range from yellow-brown to red, occurs after the introduction of physiological changes according to nutritional requirements: e.g., nitrogen sources, carbon sources, and vitamin B_12_ [31,32]. However, only β-carotene detection is mentioned in a report of carotenoids profiled in *Chlorosarcinopsis* [33]; there has been no record to-date of ketocarotenoid accumulation in this genus.

In this study, we describe a green sarcinoid alga, newly isolated from soil from western Thailand, which we identify as a new *Chlorosarcinopsis* species based on microscopic and molecular analyses. The carotenoid profiles of the green and red forms of the alga were compared together with the effect of nitrogen reduction and media volume on the enhancement of ketocarotenoid production. These findings not only represent the first report of ketocarotenoid production in sarcinoid microalgae from the genus *Chlorosarcinopsis*, but also provide insights into optimal conditions for production of canthaxanthin, an important food and feed ingredient.

## 2. Materials and Methods

### 2.1. Algal Isolation and Identification

Soil samples were collected from Pong Yoop in Ratchaburi, western Thailand, and incubated in sterile water at 25 °C under continuous white light (60 µmol·m^−2^·s^−1^) for approximately 4 weeks. Algal isolation, using a spread plate technique, was carried out on high salt minimal (HSM) medium agar [34]. Algal colonies that displayed a colony colour change from green to red after one month were selected and repeatedly re-streaked on Tris-acetate-phosphate (TAP) agar [35] until an algal axenic culture was obtained. Bright-field microscopy was carried out using an Olympus CX31 (Olympus, Tokyo, Japan) at 400× magnification for morphological studies. For molecular identification, total genomic DNA was extracted using a phenol-chloroform method [36], then polymerase chain reaction (PCR) was carried out with primer pairs used as recommended by Hall et al. [37] for *tuf*A gene (tufA.F and tufA.R), and Suutari et al. [38] and Moro et al. [39] for 18S ribosomal DNA (107F and ChloroR). PCR products were gel-purified and subjected to DNA sequencing. In order to determine the algal taxa, the obtained nucleotide sequences were analyzed using the BLAST network service [40].

### 2.2. Algal Growth and Experimental Conditions

Unless stated otherwise, all the algal samples were maintained on TAP medium at 25 °C under continuous white light (60 µmol·m^−2^·s^−1^) as an active stock. For all experiments, the algal solutions were prepared by homogenization of the fresh algal cells in liquid medium with micropestle at a concentration of 20 mg fresh weight per mL. Then a 50 µL of algal solution was used as a cell inoculum in each experimental replicate.

Algal trophic tests on solid medium; autotrophic, mixotrophic and heterotrophic growth, were tested on HSM with light, TAP with light, and TAP with no light, respectively. The nitrogen concentration effect on algal growth was investigated by increasing nitrogen concentration (in the form of NH^4+^) 2-fold from 7.48 mM (replete TAP medium) to 14.96 mM (2×) and 3-fold to 22.4 mM (3×), and decreasing it 2-fold to 3.74 mM (50% N) and 10-fold to 0.748 mM (10% N) under continuous white light of 60 µmol·m^−2^·s^−1^ at 25 °C.

A nitrogen reduction effect on algal growth and ketocarotenoid production was examined on 50% N and 10% N solid TAP medium with algal cells collected weekly for 4 weeks. To identify the effect of media volume on algal growth and ketocarotenoid production, the alga was cultured using 50% N liquid TAP medium in 15 mL, 30 mL and 60 mL in 250 mL bottles (Schott Duran, Mainz, Germany) manually shaken once a day and collected weekly for 4 weeks. Algal growth was evaluated via dry weight (mg) and the results of 7 and 28 day-old cultures were used to calculate a relative growth rate, RGR (mg·mg^−1^·d^−1^) using Equation (1) [41];

(1) $$\mathrm{RGR}=\frac{\left({\mathrm{lnW}}_{2}-{\mathrm{lnW}}_{1}\right)}{\left(t_{2}-t_{1}\right)}$$

where W_1_ and W_2_ are algal fresh weights at times t_1_ and t_2_ Quantification of canthaxanthin was analyzed at 28 days as described in the following section.

### 2.3. Pigment Extraction and Analysis

Ten milligrams of freeze-dried and ground algal powder were extracted in HPLC grade methanol, water, and chloroform (400:400:800 µL), and dried in a GeneVac Ez-2 Plus rotary evaporator (SP Scientific, Warminster, PA, USA). The crude algal extracts were separated on an Ultra High-Performance Liquid Chromatography system (Acquity™ Ultra Waters, Ltd., Borehamwood, UK) Separation used an Ethylene Bridged Hybrid (BEH C18) column (2.1 × 100 mm, 1.7 μm) with a BEH C18 VanGuard pre-column (2.1 × 50 mm, 1.7 μm). The mobile phase used was A: MeOH/H2O (50/50) and B: ACN (acetonitrile)/ethyl acetate (75:25). The gradient was 50% A:50% B for 0.5 min and then stepped to 30% A:70% B for 4.5 min, to 0% A:100% B for 2 min, back to 30% A:70% B for 1 min and to 50% A:50% B for the remaining two minutes. Column temperature was maintained at 30 °C and the temperature of samples at 8 °C. On-line scanning across the ultraviolet/visible range was performed in a continuous manner from 250 to 600 nm, using an extended wavelength photo diode array detector (Waters, Borehamwood, UK). Identification used co-chromatography and spectral properties with authentic standards. Carotenoids were identified on the basis of their absorption spectra and retention time relative to authentic standard compounds (β-carotene and xanthophylls purchased from Sigma-Aldrich except canthaxanthin, which was purified in-house from *E. coli* strains harbouring the biosynthetic genes [42]). For quantification, carotenoids were calculated from dose-response curves of standards as described in Fraser et al. [43].

### 2.4. Data Analysis

Each experiment had at least three replications and all were expressed as the mean ± SD. The significance of the variables was assessed by one-way analysis of variance (ANOVA) and post-hoc Duncan’s test with *P* < 0.05 using SPSS (IBM Corporation, New York, NY, USA).

## 3. Results

### 3.1. Isolation and Identification of the Algal Strain PY02

Several axenic cultures of microalgae were obtained as isolated green colonies growing on minimal medium following plating of the supernatant from resuspended soil samples collected from Ratchaburi province in western Thailand. Subsequently, when the isolates were transferred onto acetate-containing medium, several developed distinctive deep red colonies following one month’s incubation. One such isolate was selected for further study and was named PY02.

**Figure 1:**
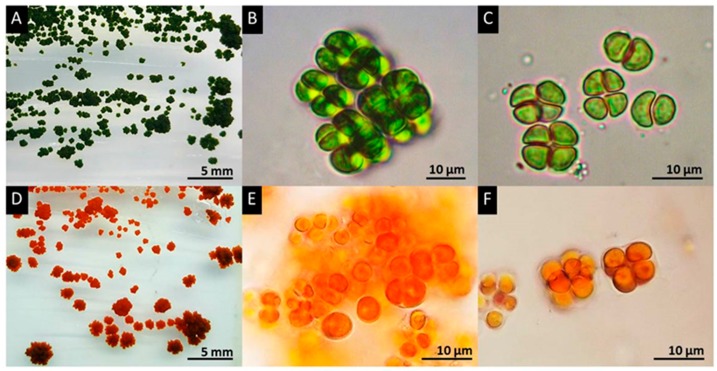
Morphologies of green and red cells of *Chlorosarcinopsis* PY02. (**A**) Green colonies on solid medium; (**B**,**E**) sarcinoid features formed by non-segregated vegetative cell division is seen in both green (**B**) and red cells (**E**); (**C**) the tetrad green cell stage; (**D**) Orange-red pigment accumulation resulting from secondary carotenoid accumulation appeared in red colonies on solid medium; (**E**) sarcinoid form in red cells; (**F**) tetrad red cells. PY02 was cultivated on TAP agar. (**A**–**C**) were taken on day 10. (**D**–**F**) were taken on day 35.

On solid medium (Figure 1A,D), PY02 forms a colony with an irregular shape, rough surface and undulating margin. Expansion of the colony was observed in both horizontal and vertical directions. Morphological investigation of PY02 under a light microscope revealed that vegetative cells are spherical when solitary with a single cell size of 4–10 µm in diameter (Figure 1). Each cell had a parietal chloroplast, a single pyrenoid, and motile zoospores with two flagella. The microscopic colony following cell division is commonly observed as three-dimensional cubic-packets of 2–8 cells typical of sarcinoid green algae. Based on this morphological information, the strain was initially identified as a member of the genus *Chlorosarcinopsis* [30,44]. Additional confirmation of this identification was achieved by molecular analysis of partial 18S ribosomal RNA (GenBank: MG825902.1) and *tuf*A (in submission) gene sequences, which supported the proposal that PY02 is a member of *Chlorosarcinopsis* (with the highest percentage identity (97%) to GenBank: KY086480.1 and HQ246367.1 for 18S ribosomal RNA and *tuf*A, respectively (BLAST on 1/02/2018, https://blast.ncbi.nlm.nih.gov/Blast.cgi). There was, however, insufficient information for discrimination at species level, and more extensive molecular studies are required.

### 3.2. Chlorosarcinopsis PY02 Is Capable of Synthesizing Ketocarotenoids

**Figure 2:**
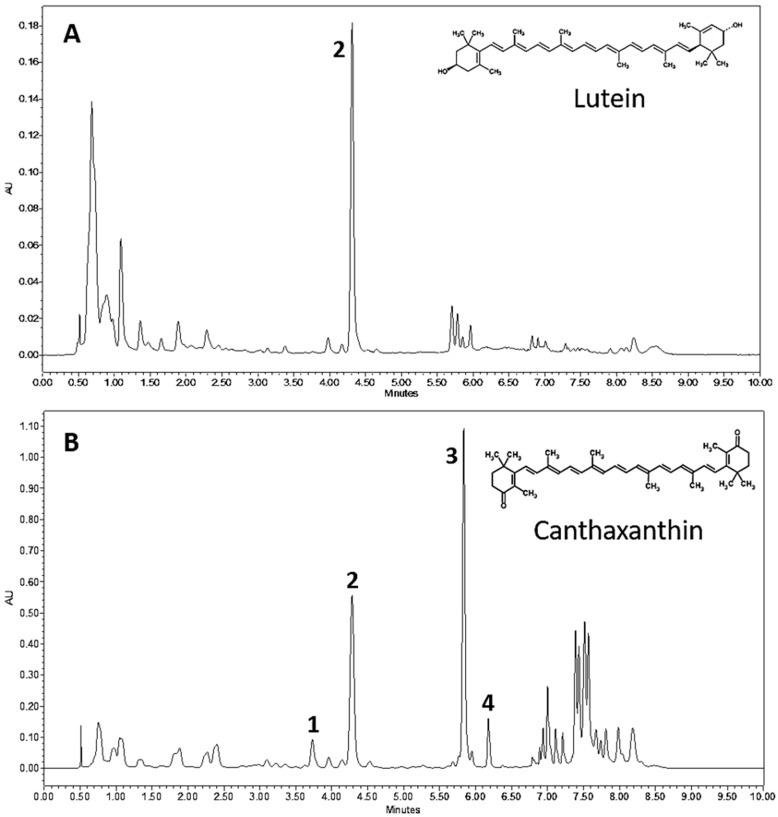
UPLC chromatograms of *Chlorosarcinopsis* PY02 extracts from green (**A**) and red (**B**) cells. Peak 1 = adonixanthin, peak 2 = lutein, peak 3 = canthaxanthin and peak 4 = 3-OH-echinenone.

In order to characterize the red pigmentation observed in PY02, the composition of carotenoid pigments in crude extracts from green and red cells were compared by ultrahigh-performance liquid chromatography (UPLC). The results are shown in Figure 2 and present the first reported evidence of an alga from the genus *Chlorosarcinopsis* having the ability to synthesize ketocarotenoids. The green and red extracts show clear differences in both pigment content and profile with lutein (peak 2) as the dominant carotenoid in green cells (Figure 2A), whereas the ketocarotenoid canthaxanthin (peak 3) is dominant in red cells (Figure 2B). Several other ketocarotenoids are also found in red cells, for example, adonixanthin (peak 1) and 3-OH-echinenone (peak 4).

### 3.3. Ketocarotenoid Accumulation in Chlorosarcinopsis PY02 Is Influenced by Growth Conditions

**Figure 3:**
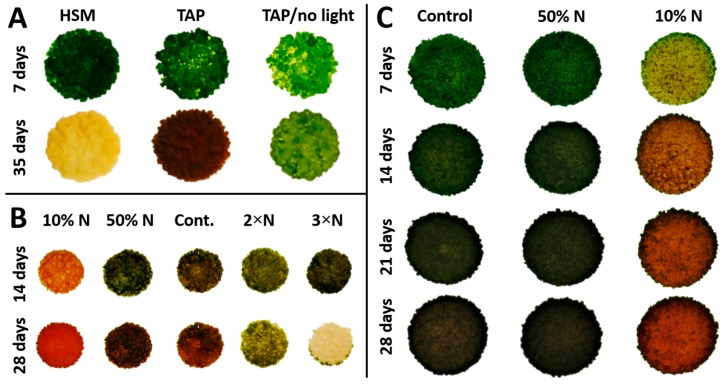
Growth pattern and nitrogen effect on mixotrophic growth of Chlorosarcinopsis PY02. (**A**) test for trophic pattern; (**B**) test for nitrogen effect on growth and carotenoid production by increasing nitrogen concentration (in the form of NH^4+^) 2-fold from 7.48 mM (replete TAP medium) to 14.96 mM and 3-fold to 22.4 mM, and decreasing it 2-fold to 3.74 mM (50% N) and 10-fold to 0.748 mM (10% N); (**C**) test for effect of nitrogen reduction on carotenoid accumulation by decreasing nitrogen concentration 2-fold to 3.74 mM (50% N) and 10-fold to 0.748 mM (10% N). Abbreviation for solid media with 1.2% agar: HSM = high salt minimal; TAP = tris-acetate-phosphate; TAP/no light = TAP with no light; 10% N = TAP with 10-fold nitrogen decrease; 50% N = TAP with 2-fold nitrogen decrease; Cont. = control replete TAP nitrogen concentration; 2×N = TAP with 2-fold nitrogen increase; 3×N = TAP with 3-fold nitrogen increase.

Studies of PY02 growth on solid medium showed that it is capable of phototrophic growth on a minimal medium (HSM), while also exhibiting mixotrophic or heterotrophic growth by utilizing acetate as an exogenous carbon source when grown on acetate-containing medium (TAP) with or without light (Figure 3A). However, a smaller colony size and fewer cells were obtained under heterotrophic growth indicating this condition is less favourable. Interestingly, mixotrophy significantly influences the accumulation of ketocarotenoids as seen by the appearance of red colonies at 35 days (Figure 3A) and confirmed with UPLC analysis. In contrast, growth in the dark did not induce the same level of carotenogenesis and photosynthetic growth on HSM medium continued only until the third week. Then the colonies gradually turned chlorotic and died at week five (Figure 3A). These studies show that secondary carotenoid accumulation in *Chlorosarcinopsis* PY02 is best achieved under mixotrophic growth conditions.

### 3.4. Nitrogen Concentration Affects Growth and Canthaxanthin Accumulation

Having established that mixotrophy is optimal for ketocarotenoid production, this growth mode was used to investigate the effect of nitrogen concentration on growth and carotenoid production. A preliminary study of the effect of five nitrogen concentrations on PY02 growth is presented in Figure 3B. At 14 days, PY02 showed growth under each condition. Nevertheless, a variation in colony colour was observed depending on the nitrogen concentration, with significant carotenogenesis seen at the lowest nitrogen level. At 28 days, unfavorable growth of PY02 was seen at elevated levels (2×N, 3×N) as sparse and pale colonies, with marked chlorosis seen at the higher level. A further study of the effect of reduced nitrogen (Figure 3C) showed that reducing the level to 50% has a minimal effect on growth and carotenogenesis, while the lowest level (10% N) results in a marked accumulation of ketocarotenoids. Based on this result, the 50% N and 10% N culture conditions were selected to quantify the effect of nitrogen deprivation on PY02 biomass and ketocarotenoid production.

**Figure 4:**
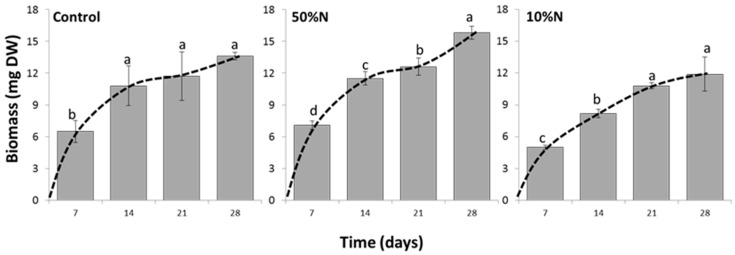
Time course of the growth in *Chlorosarcinopsis* PY02 under different nitrogen concentrations. Control = TAP with replete nitrogen concentration; 10% N = TAP with 10-fold nitrogen decrease; 50% N = TAP with 2-fold nitrogen decrease. Data was analysed by one-way ANOVA, carried out by a post-hoc Duncan’s test (n = 3, *P* < 0.05). Values are the mean from measurements of three individual samples, with the lowercase letters indicating statistical difference at *P* < 0.05 within each graph.

The growth of the alga under conditions of Control, 50% N and 10% N, as measured in biomass, are shown in Figure 4. Under all conditions, the highest biomass was obtained at week 4 (28 day-culture), although it was found that reducing nitrogen concentration delays the onset of the stationary phase. The biomass from week 2 to 4 were not statistically different in the control experiment indicating the culture entered the stationary phase at 14 days whereas under the lowest nitrogen (10% N) the culture reached this phase at 21 days. The biomass and relative growth rate (RGR; mg mg^−1^·d^−1^) of 28 day-old cultures demonstrated the highest growth in 50% N, then control and 10% N, respectively. As detailed in Table 1, the 50% N culture resulted in a statistically significant higher biomass of 15.8 ± 0.6 mg and RGR of 0.370 ± 0.005 mg· mg^−1^·d^−1^ (*P* < 0.05) indicating up to 16% higher growth than the control (biomass, 13.6 ± 0.4 mg and RGR, 0.357 ± 0.001 mg·mg^−1^·d^−1^). Although the lowest biomass and RGR were obtained from the 10% N culture with a biomass of 11.9 ± 1.6 mg and RGR of 0.353 ± 0.004 mg·mg^−1^·d^−1^, there was no significant difference in the RGRs and biomasses between the 10% N culture and the control (Table 1).

A reduction of nitrogen concentration plays an important role in the induction of canthaxanthin accumulation in PY02 alga as seen in Figure 3C. A 10% N culture showed a distinctive change in colony colour from 7 days of a greenish-yellow to a brownish-red over time. Additionally, canthaxanthin was detected by UPLC in a 7 day-old culture whereas none was found in 50% N and control cultures. The canthaxanthin content of all three growth conditions was analyzed at 28 days (stationary phase of 10% N culture). Although no noticeable difference of colony colour was visibly detected between the 50% N culture and the control (Figure 3C), the quantification of canthaxanthin content in 28 day-old cultures indicated a 3-fold elevation of canthaxanthin content from 520 ± 178.2 to 1504 ± 108.9 and 1427 ± 255.9 µg·gDW^−1^ in 50% N and 10% N cultures, respectively (Table 1). Within the genus *Chlorosarcinopsis*, there are no previous reports of an ability to synthesize ketocarotenoids. However, compared with reports of other microalgae, canthaxanthin content in PY02 is slightly lower than the findings in *Dactylococcus* (approximately 1700 µg·gDW^−1^) in which the canthaxanthin production was induced by nitrate starvation, NaCl addition of 7.5 g·L^−1^, and variation of light intensity [24].

The faster change of PY02 colony colour under the lowest nitrogen concentration is presumed to occur in response to nutrient starvation stress [45,46,47]. However, as the results of this experiment reveal, the 50% N condition provides the highest potential production of PY02. This condition enhances both growth and canthaxanthin production since initially nutrients including nitrogen are not limited allowing rapid growth in the early stages. After depletion of the available nitrogen, the alga becomes stressed and the C/N ratio increases, thereby enhancing secondary carotenoid accumulation.

### 3.5. The Volume of Culture Medium Influences Growth and Canthaxanthin Production in PY02

**Figure 5:**
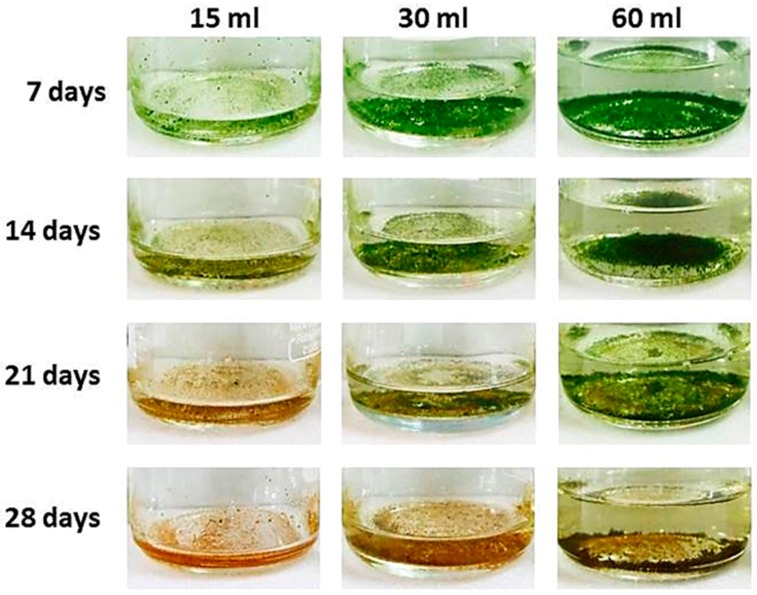
Effect of media volume on *Chlorosarcinopsis* PY02 growth and cell colour. This experiment was conducted using 50% N TAP liquid medium with initial volumes of 15, 30 and 60 mL in 250 mL Schott Duran bottles.

As shown in Figure 5, PY02 tends to grow in liquid culture as clumps or flocs that readily collect as a layer at the bottom of the flask. In order to determine if the volume of medium influences these flocs, an experiment was conducted under the optimum nitrogen concentration of 50% reduction using a range of volumes (15 mL, 30 mL, 60 mL). As shown in Table 2, both the significantly highest biomass and RGR (*P* < 0.05) were from the 60 mL culture, then the 30 mL and 15 mL, respectively. On the other hand, the significantly highest dry weight per mL (*P* < 0.05) was found in the 15 mL culture (1.580 ± 0.05 mg·mL^−1^) then the 30 mL (1.260 ± 0.04 mg·mL^−1^) and 60 mL (1.040 ± 0.02 mg·mL^−1^) respectively.

Regarding the effect of media volume on canthaxanthin content (Table 2), the 15 mL culture had a significantly higher canthaxanthin content (*P* < 0.05) of 898.2 ± 101 µg·gDW^−1^ and higher canthaxanthin production of 1.421 ± 0.228 µg·mL^−1^ than the 30 mL (576.8 ± 96 µg·gDW^−1^ and 0.735 ± 0.174 µg·mL^−1^) and 60 mL cultures (335.3 ± 64 µg·gDW^−1^ and 0.348 ± 0.065 µg·mL^−1^). Additionally, the 15 mL volume induced the fastest change in cell colour as seen in Figure 5.

In addition, UPLC analysis of crude extract in this experiment showed that β-carotene was detected in the 7 and 14 day-old cultures, but none after this whereas canthaxanthin continued to increase. The formation of products from β-carotene is more likely attributed to the metabolic transition to ketocarotenoid production and their role in combating antioxidative stress under changing environmental cultivation conditions [48,49,50].

## 4. Discussion

Bioprospecting for novel microalgal species offers new opportunities for sustainable sources of commercially valuable compounds. These include carotenoids that have applications as food and cosmetic colourants, and as antioxidants [51]. The chlorophyte isolate described here is identified as a member of the genus *Chlorosarcinopsis* based on morphological and molecular analysis, although classification at the species level requires further molecular characterization such as acquiring more DNA sequence information particularly from the chloroplast genome [51], and also examination of subcellular structures such as flagella and stigma position in zoospores [32]. The detection of ketocarotenoids in PY02 represents the first report for a *Chlorosarcinopsis* species, and not only offers a new biological source of these compounds but will also be of value in basic research aimed at understanding the complexity of carotenoid biosynthetic amongst algae [28].

The discovery that PY02 is capable of utilizing exogenous acetate as a carbon source and can, therefore, grow under mixotrophic conditions enhances the potential of this species for carotenoid production. Mixotrophic cultivation takes advantage of an alga’s ability to utilize an exogenous carbon source and perform photosynthesis simultaneously and offers an enhancement of biomass production over photoautotrophic cultivation, as reported for green microalgae such as *Chlorella* spp. [52,53]. The finding that PY02 also accumulates ketocarotenoids under these conditions correlates with previous studies in *Haematococcus* which similarly showed that the supplement of acetate induces ketocarotenoid accumulation [19,28,54]. Additional studies with other carbon sources such as pyruvate might further improve growth and accumulation in PY02 [19].

**Figure 6:**
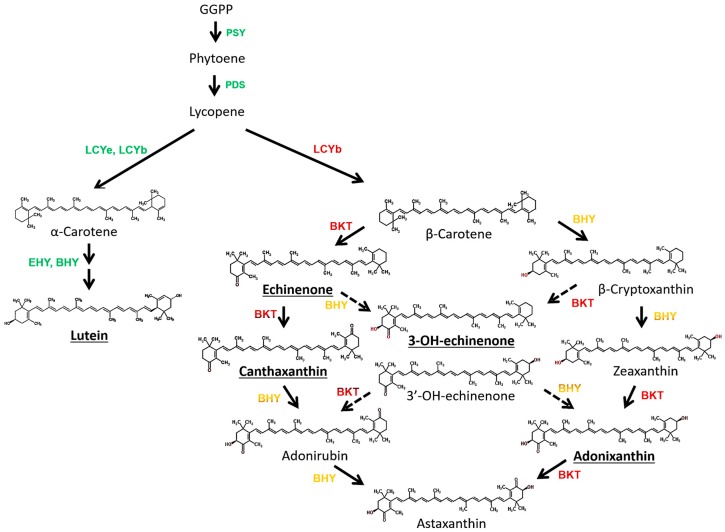
Possible carotenoid biosynthesis pathway in *Chlorosarcinopsis* PY02. Ketocarotenoids in bold and underlined were detected in this study. The carotenogenic enzymes involved in various reactions are indicated. The enzymes written in green signify the pathway in green cells whereas those written in red and yellow involve ketocarotenoid biosynthesis pathway under stress conditions. PSY: phytoene synthase; PDS: phytoene desaturase; LCYe: lycopene ε-cyclase; LCYb: lycopene β-cyclase; EHY: ε-ring hydroxylase BHY: β-ring hydroxylase; BKT: β-carotene ketolase.

The detection of 3-OH-echinenone and adonixanthin in PY02 supports the assumption that this alga harbours both β-carotene ketolase (BKT), which introduces keto groups to the β-ionone rings of β-carotene [55,56], and β-carotene hydroxylase (BHY) which introduces a hydroxyl group to the β-ionone rings [57]. However, given that canthaxanthin (derived from β-carotene by the addition of two ketone groups) is the major ketocarotenoid in PY02, this indicates that the ketolase is more active than the hydroxylase since both enzymes compete for β-carotene as substrate [58]. Although astaxanthin (derived from β-carotene by the addition of two ketone groups and two hydroxyl groups) was not detected in the algal extracts, the evidence of BKT and BHY activities through the detectable amounts of 3-OH-echinenone and adonixanthin (products with single ketone and hydroxyl groups, or one ketone group and two hydroxyls, respectively) indicated the possibility that PY02 is able to synthesize the highly valued astaxanthin. The possible pathways from β-carotene to astaxanthin are shown in Figure 6.

Nitrogen is quantitatively the most important element contributing to the dry matter of microalgal cells. Hence, when PY02 is grown in nitrogen deprived conditions, the available nitrogen is not enough to sustain cellular metabolism, resulting in a reduced biomass. This effect is similar to the results that have been reported in several microalgae [18,59], although the optimum nitrogen concentration for growth differs depending on the species [60,61]. For PY02, 50% of the nitrogen concentration in standard TAP medium appears close to the optimum, with higher levels (e.g., 300%) compromising growth and survival. Under mixotrophic growth condition, we suspect that the reduced nitrogen concentration resulted in more rapid depletion and subsequent stress. However, it is worth noting that algal biomass accumulation continues for several more days [62]. A reduction of nitrogen concentration is seen to induce canthaxanthin accumulation in PY02 similar to the phenomena that have been reported in several other microalgae [18,59] including the commercial species *Haematococcus pluvialis*. Moreover, the addition of acetate in combination with nitrogen deprivation further compounds the high C/N ratio that could be triggering the ketocarotenoid accumulation. A similar effect of a high C/N ratio has been reported in several microalgae such as *Haematococcus* [63,64] and *Chlorella* [65].

Two interesting features of PY02 are: (i) its readiness to aggregate into clumps or flocs in liquid medium, resulting in the algae readily settling on the bottom of the culture flask, and (ii) the effect of culture volume on cell growth and colour. The aggregation is most likely caused by the extracellular gelatinous matrix that is produced in order to organize a group of cells into a sarcinoid or cubic packet-forming assemblage [29,66]. Although the heterogeneous dispersal of the algal clumps in liquid medium hindered the study of specific growth rate (e.g., by cell counting or spectrophotometer measuring), this natural flocculation might help to reduce the dewatering expense that can account for more than 30% of the entire bioprocess costs [67,68]. A video showing natural settling of a PY02 suspension and complete clearing of the medium in less than 20 min is presented as Supplementary Video S1. The increased biomass per unit volume observed in the lower volume cultures (Figure 5) might reflect more efficient aeration of the culture during mixotrophic growth [69], whereas the earlier onset of carotenogenesis is probably due to more rapid exhaustion of nutrients in the smaller volume. Careful consideration of these factors would therefore be key to the successful scale-up of PY02 cultivation for commercial ketocarotenoid production.

## 5. Conclusions

Microalgae are a valuable source of natural carotenoids and offer a sustainable production advantage. In order to enhance carotenoid accumulation, microalgae require stress conditions that up-regulate carotenoid biosynthesis. This study shows that reduction of nitrogen in the media significantly stimulates carotenoid production in a newly isolated microalga (PY02) that we have classified as a species of *Chlorosarcinospsis*. The high value ketocarotenoid canthaxanthin is reported for the first time in this chlorophyte and shown to be the major pigment in response to nitrogen starvation stress. Additionally, two interesting features of PY02 are described: (i) its readiness to aggregate into clumps or flocs in liquid medium, resulting in the algae readily settling on the bottom of the culture flask, and (ii) the effect of culture volume on cell growth and colour. The findings from this study not only provide information on canthaxanthin accumulation in *Chlorosarcinospsis* but also offer a new natural source for carotenoid production.

## Figures and Tables

**Table 1:** Effects of nitrogen deprivation on algal growth, biomass and canthaxanthin content at 28 days.

Initial N Conc. ^1^	Biomass (mg)	RGR ^2^ (mg·mg^−1^·d^−1^)	CA Content ^3^ (µg·g^−1^ DW)
Control	13.6 ± 0.4 ^a^	0.357 ± 0.001 ^a^	520.9 ± 178.2 ^a^
50% N	15.8 ± 0.6 ^b^	0.370 ± 0.005 ^b^	1504.2 ± 108.9 ^b^
10% N	11.9 ± 1.6 ^a^	0.353 ± 0.004 ^a^	1426.7 ± 255.9 ^b^

^1^ Initial nitrogen concentration; ^2^ Relative growth rate; ^3^ Canthaxanthin content and DW is dry weight. Data was analysed by one-way ANOVA, carried out by post-hoc Duncan’s test (n = 3, *P* < 0.05). Values are the mean from measurements of three individual samples. Superscript letters indicate statistical difference at *P* < 0.05 of each parameter.

**Table 2:** Effects of media volume on algal growth, biomass, canthaxanthin content and production at 28 days.

Media Volume (mL)	Biomass (mg)	RGR ^1^ (mg·mg^−1^·d^−1^)	Dry Weight per mL (mg·mL^−1^)	CA Content ^2^ (µg·gDW^−1^)	CA Production ^3^ (µg·mL^−1^)
15	23.60 ± 0.60 ^c^	0.378 ± 0.002 ^c^	1.580 ± 0.05 ^a^	898.2 ± 101 ^a^	1.421 ± 0.228 ^a^
30	38.00 ± 1.14 ^b^	0.395 ± 0.002 ^b^	1.260 ± 0.04 ^b^	576.8 ± 96 ^b^	0.735 ± 0.174 ^b^
60	61.60 ± 1.81 ^a^	0.412 ± 0.002 ^a^	1.040 ± 0.02 ^c^	335.3 ± 64 ^c^	0.348 ± 0.065 ^c^

^1^ Relative growth rate; ^2^ Canthaxanthin content; ^3^ Canthaxanthin production. Data was analysed by one-way ANOVA, carried out by post-hoc Duncan’s test (n = 3, *P* < 0.05). Values are the mean from measurements of three individual samples. Superscript letters indicate statistical difference at *P* < 0.05 of each parameter.

## Supplementary Materials

The following is available online at http://www.mdpi.com/2079-7737/7/1/17/s1: Video S1: PY02 harvesting.
